# Diagnosis and Management of Traumatic Spondyloptosis: A Series of 30 Cases

**DOI:** 10.7759/cureus.105703

**Published:** 2026-03-23

**Authors:** Guillermo I Ladewig Bernaldez, Hector A Perea Gutierrez, Juan M Ocampo Godinez, Gerson Gomez Flores, Alejandro Miguel Zambrano, Christian J Sandoval Ramírez, Edith Oropeza Oropeza, Manuel Dufoo Olvera

**Affiliations:** 1 Spine Surgery, Clínica de Columna "Dr. Manuel Dufoo Olvera" IMSS Bienestar, Ciudad de México, MEX; 2 Immunology, University College London, London, GBR; 3 Spine Surgery, Hospital Angeles Pedregal, Ciudad de México, MEX

**Keywords:** accidental falls, asia, high-energy trauma, physically disabled, spinal cord injuries, spondyloptosis, trauma centers

## Abstract

Introduction: Traumatic spondylolisthesis is a severe injury involving 100% displacement of one vertebral body over another in the coronal or sagittal plane, caused by the complete disruption of spinal stabilizing elements due to high-energy trauma. This injury often compromises vital organ structures, requiring prompt management to reduce morbidity and mortality.

Materials and methods: A case series was conducted at the Spine Clinic "Dr. Manuel Dufoo Olvera" IMSS Bienestar from February 1, 2015, to June 29, 2025. Data included sex, age, injury mechanism, vertebral segment, associated injuries, neurological deficits, hospital stay, and treatment.

Results: 30 cases (100%) were reported: 23 men (76.67%) and 7 women (23.33%), with ages ranging from 18 to 67 years (mean: 35). Injury mechanisms were falls from height (21 cases, 70.97%) and motor vehicle accidents (9 cases, 29.03%). Affected segments: cervical spine, 4 cases (13.33%), cervicothoracic junction, 3 cases (10%), thoracic spine, 16 cases (53.33%), thoracolumbar junction, 4 cases (13.33%), and lumbar spine, 3 cases (10%). Neurological deficits: ASIA A 20 cases (66.67%), ASIA B 2 cases (6.67%), ASIA C 1 case (3.33%), and ASIA D 7 cases (23.33%). Associated injuries: thoracic trauma 16 cases (52.17%), head trauma 8 cases (30.43%), extremity trauma 3 cases (8.70%), and abdominal trauma 3 cases (8.70%). Hospital stay ranged from 7 to 105 days (mean: 37.89). A total of 27 cases (89.25%) survived, while 3 cases (10.75%) died.

Discussion: Findings align with other reports regarding male predominance, injury mechanisms, and neurological deficits. Key differences included the affected age group and the lack of prior documentation on hospital stay duration.

Conclusion: Traumatic spondyloptosis is a devastating injury requiring comprehensive evaluation and strict multidisciplinary management to reduce morbidity, mortality, and hospital stay and facilitate social reintegration.

## Introduction

Traumatic spondyloptosis is the most severe injury that can occur in the spine. It is defined as the complete slippage of one vertebral body over another by 100% or more, either anteriorly, posteriorly, or laterally over the caudal vertebra, in either the sagittal or coronal plane. This injury can occur in any anatomical segment of the spine and involves disruption of all three columns, leading to complete instability [1,2]. It is a lesion secondary to high-energy trauma, such as falls from heights, traffic accidents, and injuries related to the operation of heavy machinery.

This type of injury is generally associated with other life-threatening conditions. In traumatic spondyloptosis of the cervical spine, it is necessary to rule out the presence of traumatic brain injury (fractures, hematomas) [3-5]. At the thoracic level, the most unusual location due to the stiffness of this segment provided by the sternum, ligaments, and costal arches, it is essential to rule out blunt chest trauma (flail chest, cardiac contusion, hemothorax, pneumothorax, pulmonary contusion) [6]. For the lumbar spine, pre-existing pathology and blunt abdominal trauma (injury to solid and/or hollow organs) must be ruled out. It is also important not to overlook associated injuries to the pelvis and appendicular skeleton. Therefore, initial management must adhere to protocols for polytrauma patients (Hannover and ATLS) [7-9].

Despite the poor prognosis of this condition, there are cases in which no neurological deficit is present. It is important to understand that the level of spinal cord injury is associated with patient morbidity and mortality. There is limited information on this type of injury in international literature, with the majority being case reports. For this reason, we present the following case series from the Spine Clinic "Dr. Manuel Dufoo Olvera" IMSS Bienestar.

## Materials and methods

The current study is a case series conducted at the Spine Clinic "Dr. Manuel Dufoo Olvera" IMSS Bienestar from February 1st, 2015, to June 29th, 2025. Thirty cases of traumatic spondyloptosis were analyzed, with the following demographic aspects reviewed: sex, age, injury mechanism, affected region of the spine, associated injuries, length of hospital stay (in days), the therapeutic management provided, and spinal cord injury according to the ASIA (American Spinal Injury Association) Impairment Scale [10] with its categories: ASIA A: Complete. No sensory or motor function is preserved in the sacral segments S4-S5. ASIA B: Incomplete. Sensory but not motor function is preserved below the neurological level and extends through the sacral segments S4-S5. ASIA C: Incomplete. Motor function is preserved below the neurological level, and the majority of key muscles below the neurological level have a muscle grade of less than 3. ASIA D: Incomplete. Motor function is preserved below the neurological level, and the majority of key muscles below the neurological level have a muscle grade greater than or equal to 3. ASIA E: Normal. Sensory and motor function is normal [10]. The data were collected retrospectively from medical records. The inclusion criteria were that the patient had the diagnosis of traumatic spondyloptosis; the exclusion criteria were the absence of the diagnosis. The primary outcomes of the study included neurological status according to the ASIA Impairment Scale, in-hospital mortality, and length of hospital stay. The secondary outcomes of the study included demographic characteristics, mechanism of injury, affected spinal segment, associated injuries, and type of therapeutic management.

Data analysis

The data was collected retrospectively from medical records and entered into a structured database for analysis. Descriptive statistical analysis was performed given the observational case series design and absence of a comparison group.

Descriptive statistics

Categorical variables (sex, mechanism of injury, affected spinal segment, ASIA grade, associated injuries, and survival status) were summarized using absolute frequencies (n) and percentages (%). Continuous variables (age and length of hospital stay) were summarized using minimum and maximum values and mean (average).

Distribution analysis

Age and length of hospital stay were evaluated descriptively due to the small sample size (n = 30). Given the limited number of cases, no formal normality testing was required for inferential purposes.

Outcome assessment

Neurological status was categorized according to the ASIA Impairment Scale (A-E) and reported as proportions. The mortality rate was calculated as the percentage of in-hospital deaths relative to the total sample. Length of hospital stay (LOS) was analyzed as a continuous variable to assess morbidity burden.

Comparative context

A narrative comparison was conducted between the findings of this series and previously published reports without formal statistical hypothesis testing due to a lack of raw comparative datasets, differences in study design, and a small sample size.

Survival analysis

The primary survival endpoint was in-hospital mortality. Survival time was defined as the number of days from hospital admission to death (event) or hospital discharge (event). We used the GraphPad Prism 8.4.3 program for the statistical analysis.

## Results

Significant cases

Case 1

A 44-year-old female patient with no chronic degenerative diseases. Her condition began after suffering a fall from approximately 4 meters in height, impacting the lateral region of the lumbar spine and her head, resulting in loss of consciousness and the onset of generalized tonic-clonic seizures lasting 4 minutes. She was attended by paramedics and transferred to a general hospital, where she received initial management in the emergency room and was subsequently admitted to the intensive care unit with advanced ventilatory support.

A diagnosis of moderate traumatic brain injury, according to the WHO, was made, with bilateral fronto-parietal contusions that did not require surgical management by the neurosurgery service. She was therefore discharged and transferred to our care center. Upon arrival, she was on ventilatory support with isochoric pupils and hypoventilated lung fields.

A neurological window was performed, revealing the following in the physical examination: Normal C5-C6-C7 osteotendinous reflexes are present, sensation is preserved down to T6, abdominocutaneous reflexes are absent, anal tone is abolished, there is an absence of the clitoridoanal reflex, L4 and S1 osteotendinous reflexes are absent, and lower limb strength from L2 distally is 0/5 on the Medical Research Council scale for muscle strength (MRC scale) [11].

**Figure 1:**
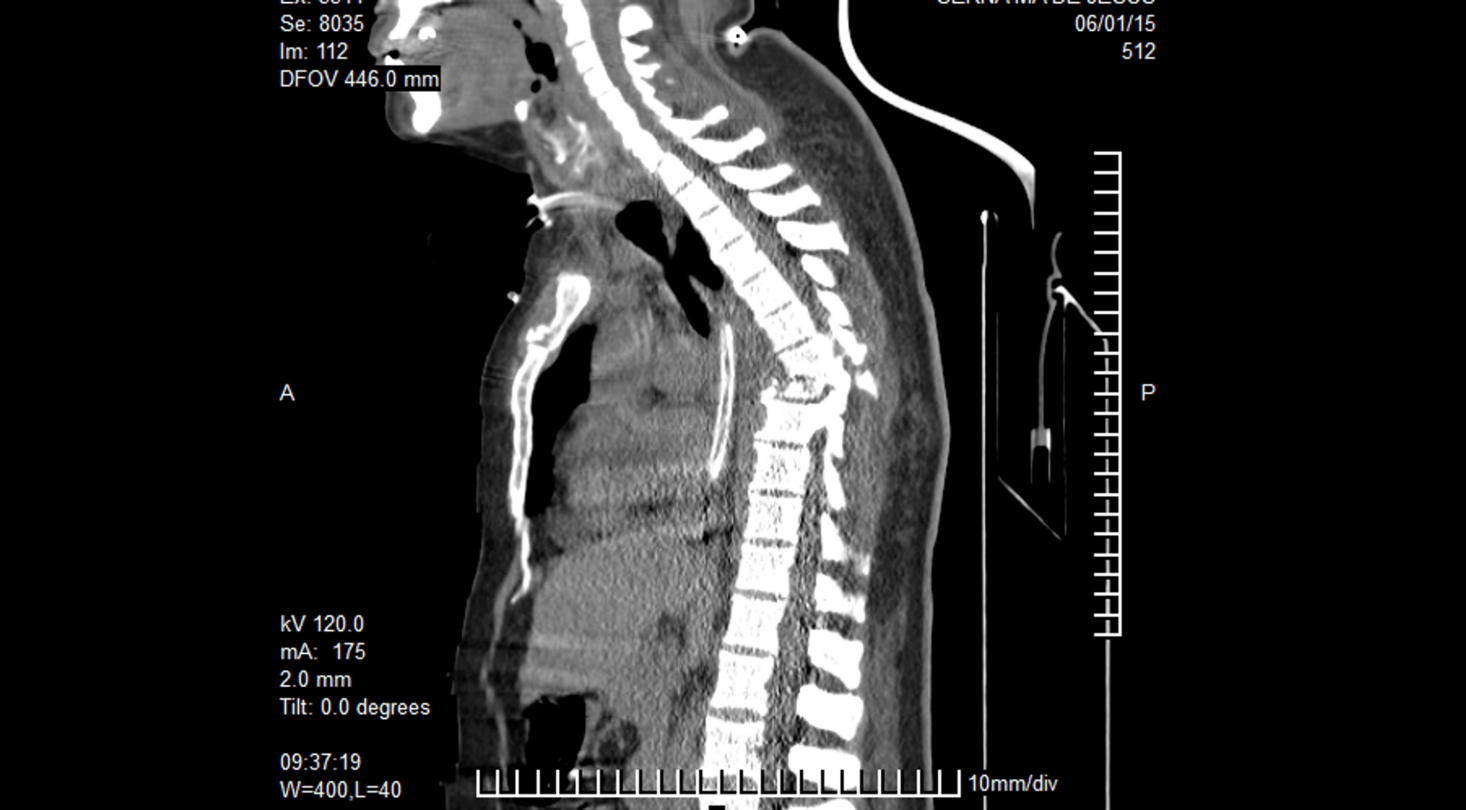
Case 1 CT scan midline sagittal cut image showing loss of continuity of the vertebral column at the level of the T6-T7 vertebrae.

**Figure 2:**
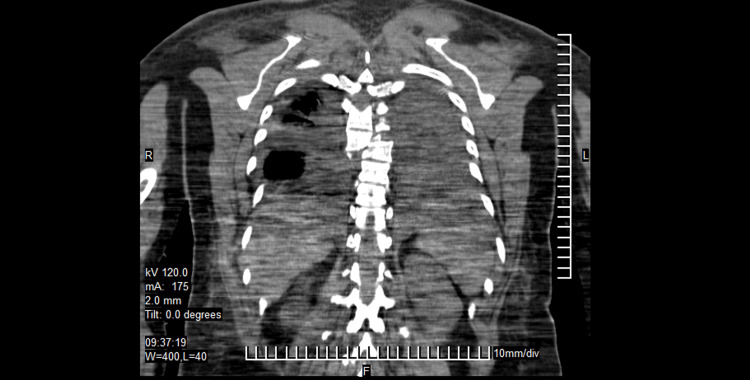
Case 1 CT scan coronal cut showing the lateral displacement of the vertebral bodies to the right and bilateral hemothorax, predominantly on the left side.

**Figure 3:**
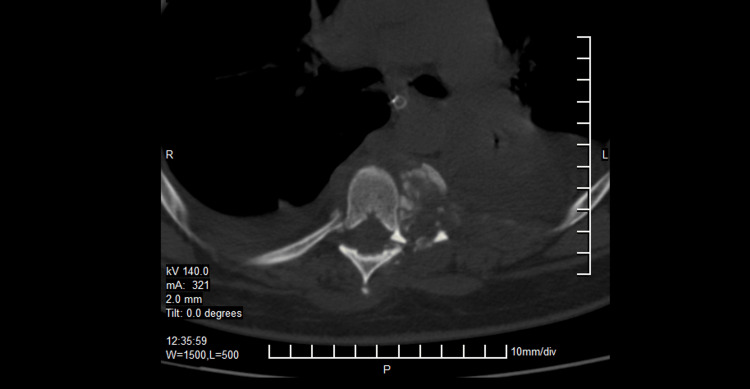
Case 1 CT scan axial cut showing the lateral displacement of the bodies and the loss of the relationship between the vertebral body and the spinal canal.

A CT scan revealed pulmonary hemorrhages and bilateral hemothorax (20% on the right and 50% on the left), along with right lateral spondyloptosis of T6 over T7 (Figures 1-3). The patient was diagnosed with a spinal cord injury, classified as ASIA.

The patient was readmitted to the intensive care unit to continue advanced ventilatory management. During her stay in the ICU, the hemothorax was evacuated with pleurostomy tubes, and a tracheostomy was performed due to prolonged orotracheal intubation.

**Figure 4:**
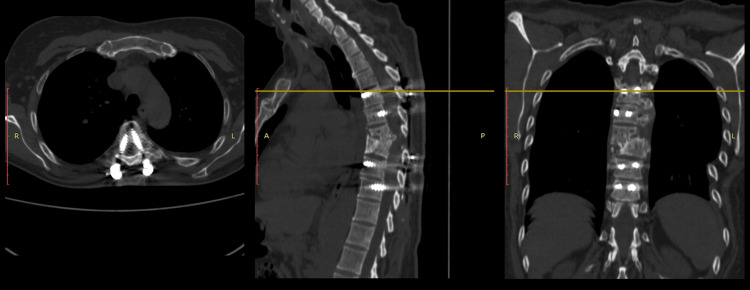
Case 1 CT scan post-surgical images of the T4-T5-T8-T9 transpedicular fixation in the axial, coronal, and sagittal planes.

The patient underwent surgery seven weeks after her injury, after the resolution of the associated conditions related to her illness. An open reduction and transpedicular fixation of T4-T5-T8-T9 was performed, along with a T6-T7 interbody fusion (Figure 4). She was discharged home 12 weeks after the onset of her condition.

Case 2

A 45-year-old male patient with no chronic degenerative diseases. His condition began while riding as a passenger in the back seat of a car; he was intoxicated and not wearing a safety device. He was ejected from the vehicle upon impact with a retaining wall, resulting in loss of motor and sensory function in all four extremities and transient loss of consciousness.

He was attended in the emergency department, where a diagnosis of moderate traumatic brain injury was made. Upon arrival at the ER, the male patient was conscious and oriented. He presented with a rigid neck, painful upon palpation.

Upper extremity strength was C4-C5-C6 5/5 bilaterally, C7 3/5 bilaterally, and C8 1/5 bilaterally on the MRC scale. Sensation was preserved down to T4, with anesthesia from T5 distally. Anal sphincter tone was absent, and the bulbocavernosus reflex was absent.

Upper extremity osteotendinous reflexes were present at C5 and C6 and abolished at C7. In the lower extremities, reflexes were absent at L4 and S1.

**Figure 5:**
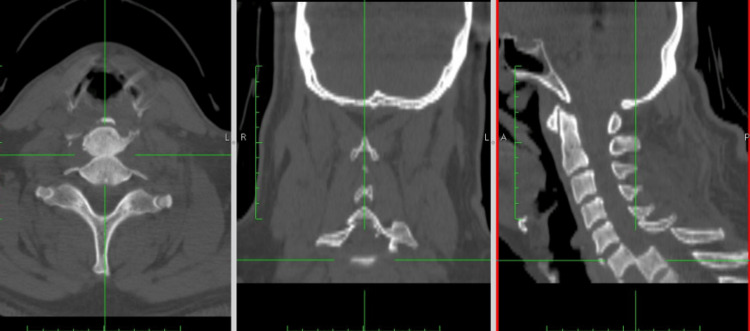
Case 2 CT scan of the C6-C7 injury in axial, coronal, and sagittal views.

Protocol imaging studies performed in the emergency department revealed a spondyloptosis of the C6 vertebra over C7 with an ASIA A spinal cord injury (Figure 5).

**Figure 6:**
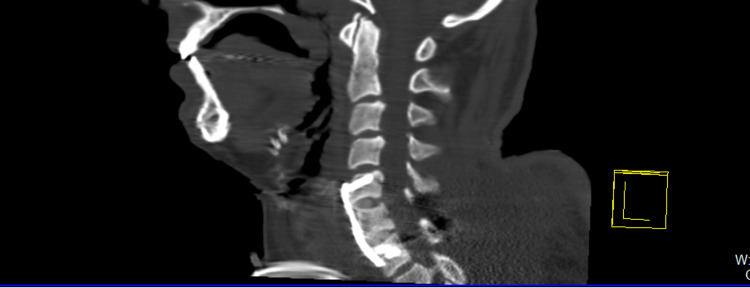
Case 2 CT scan post-surgical sagittal views of the anterior and posterior fixation from C5 to C7, showing the anterior cervical plate, intersomatic tricortical graft, and transfacetary fixation.

Gardner-Wells tongs were applied in the emergency department. Surgical management was provided, which included stabilization via posterior reduction and stabilization with C6-C7 transfacetary screws and two rods. Anterior reconstruction was performed via C5-C6 and C6-C7 discectomy, interbody arthrodesis at the same levels, autologous bone graft harvesting from the iliac crest, and anterior plate stabilization from C5 to C7 (Figure 6).

The patient had a torpid recovery due to nosocomial pneumonia caused by *Pseudomonas Aeruginosa* and died 14 weeks after admission.

Case 3

A 67-year-old male patient with a history of diabetes mellitus and hypertension. His current condition began secondary to a traffic accident involving a rollover. He was riding as a front-seat passenger and sustained impacts to the head and thorax, resulting in a transient loss of consciousness.

He was assisted by emergency services and transferred to a general hospital, where he was evaluated by the neurosurgery service, which ruled out surgical pathology. He was subsequently transferred to our center.

Upon arrival, the male patient was conscious and oriented, with a sutured wound present in the left parietal region. Pupils showed normal reflexes. The neck had no apparent deformities. The thorax had symmetric expansion and palpation, with bilateral vesicular breath sounds present. The abdomen was globose, soft, and depressible, with no signs of peritoneal irritation. The pelvis was stable both rotationally and vertically. Upper and lower extremities were anatomically intact.

The neurological examination revealed strength in C4-C5-C6-C7 at 5/5 on the MRC scale bilaterally. C5-C6-C7 osteotendinous reflexes were normal bilaterally. The upper abdominocutaneous reflex was present; the middle and lower were absent. The last sensory level was T8, with hypoesthesia from T9 to T11, and anesthesia from T12 distally. Anal tone was absent. The bulbocavernosus reflex was absent. The cremasteric reflex was absent. Lower extremity strength (L2-L3-L4-L5-S1) was 0/5 bilaterally. L4 and S1 osteotendinous reflexes were absent bilaterally.

**Figure 7:**
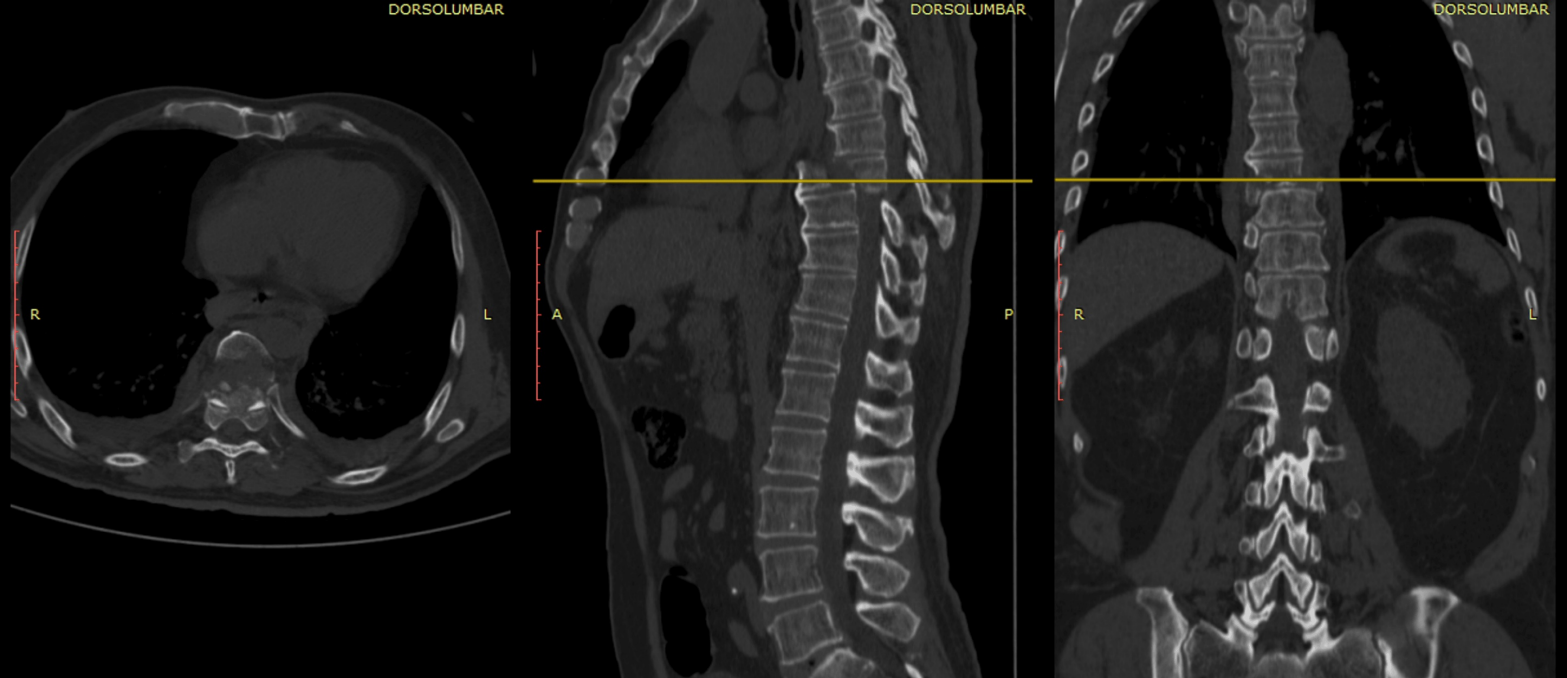
Case 3 CT scan of the initial injury and the displacement of the T8 body over T9 are visible in axial, coronal, and sagittal views.

Imaging studies (plain X-rays and CT scan) revealed a pulmonary contusion, in addition to a traumatic posterior spondyloptosis of the T8 vertebral body over T9 (Figure 7).

Associated injuries were managed. He underwent surgery three weeks after the onset of his condition, receiving short-segment posterior transpedicular instrumentation from T8 to T10 and posterolateral arthrodesis, with a plan for a second surgical stage involving a transthoracic anterior spondylodesis.

**Figure 8:**
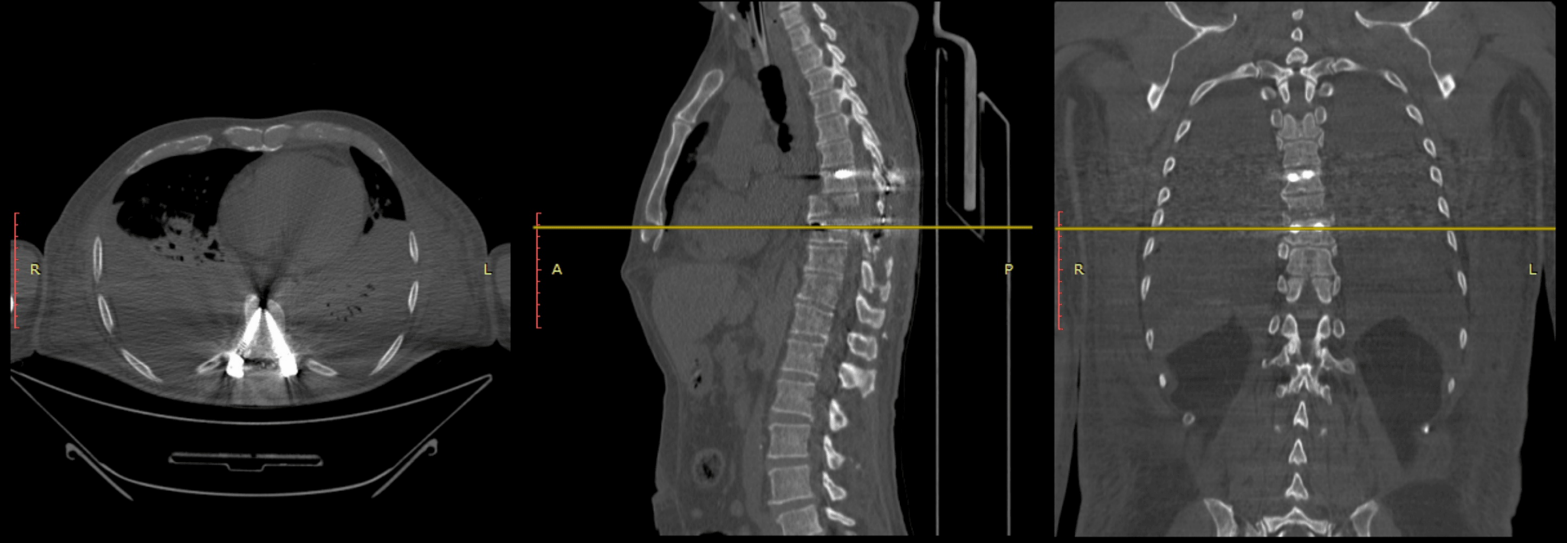
Case 3 CT scan of postoperative follow-up images showing the short-segment T8-T10 transpedicular instrumentation and the presence of bilateral hemothorax (70%).

However, during his postoperative stay, his evolution was torpid. He developed bilateral hemothorax (Figure 8) and nosocomial pneumonia caused by *Pseudomonas aeruginosa* and *Staphylococcus haemolyticus*. He died six weeks after the onset of his condition.

Case 4

A 50-year-old male patient was involved in a motor vehicle accident while driving under the influence of alcohol. He sustained impacts to the head, thorax, and upper extremities without loss of consciousness. He was treated and transferred to a general hospital, where he received initial emergency management, remaining for approximately 24 hours before being transferred to our center.

**Figure 9:**
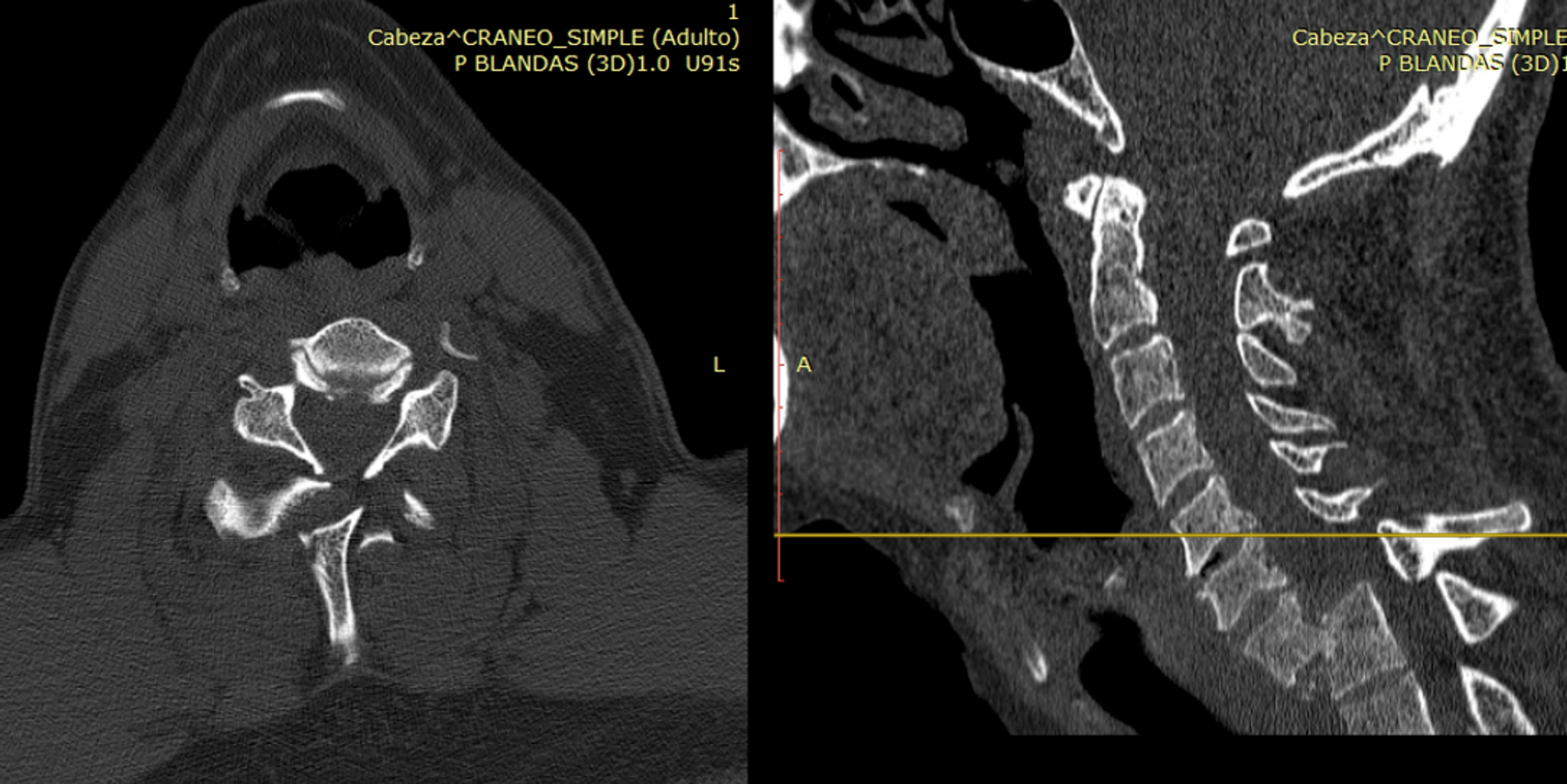
Case 4 CT scan axial and sagittal slices showing the injury of the C7 vertebral body over T1.

Diagnoses included traumatic brain injury, diaphyseal fracture of the left humerus, and traumatic spondyloptosis of C7-T1 with ASIA D spinal cord injury (Figure 9). Initial physical examination revealed frontal and periorbital edema and ecchymosis. The neck was rigid with pain on palpation in the anterior and posterior regions. The thorax had intact bilateral vesicular breath sounds with symmetric expansion and palpation, with no percussion abnormalities in the lung fields. The abdomen was soft and depressible without anatomical alterations or signs of peritoneal irritation. The left upper extremity showed longitudinal shortening, while the right upper extremity was anatomically intact. Both lower extremities were anatomically intact bilaterally.

Neurological examination revealed hypoesthesia in the C7 dermatome of the left upper extremity, as well as 3/5 strength in the left C6-C7-T1 myotomes. Right upper extremity strength was 5/5 on the MRC scale in all myotomes. Osteotendinous reflexes were present in C5-C6-C7 of the right upper extremity and not assessable in the left. Upper, middle, and lower abdominocutaneous reflexes were present. Anal sphincter tone was preserved, with the presence of the bulbocavernosus reflex and cremasteric reflex. Lower extremity strength was 5/5 on the MRC scale from L2 distally bilaterally, with sensation preserved in all dermatomes. L4 and S1 osteotendinous reflexes were present bilaterally.

Upon arrival, the patient experienced two episodes of psychomotor agitation, predominantly nocturnal. Gardner-Wells tongs were applied for cervical immobilization. Subsequently, a CT angiography of the head and cervical spine was performed to rule out vertebral artery thrombosis.

**Figure 10:**
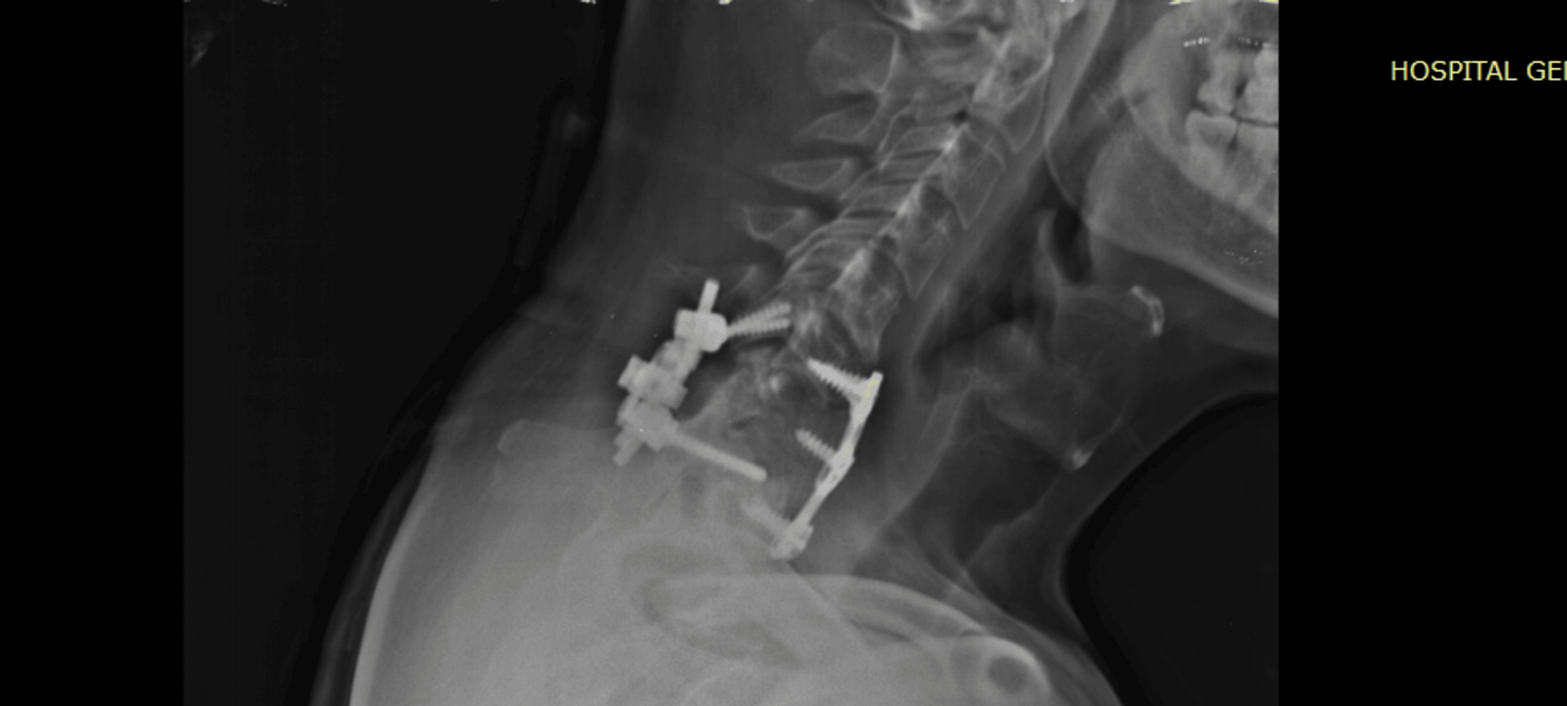
Case 4 post OP lateral X-ray of the cervical spine.

**Figure 11:**
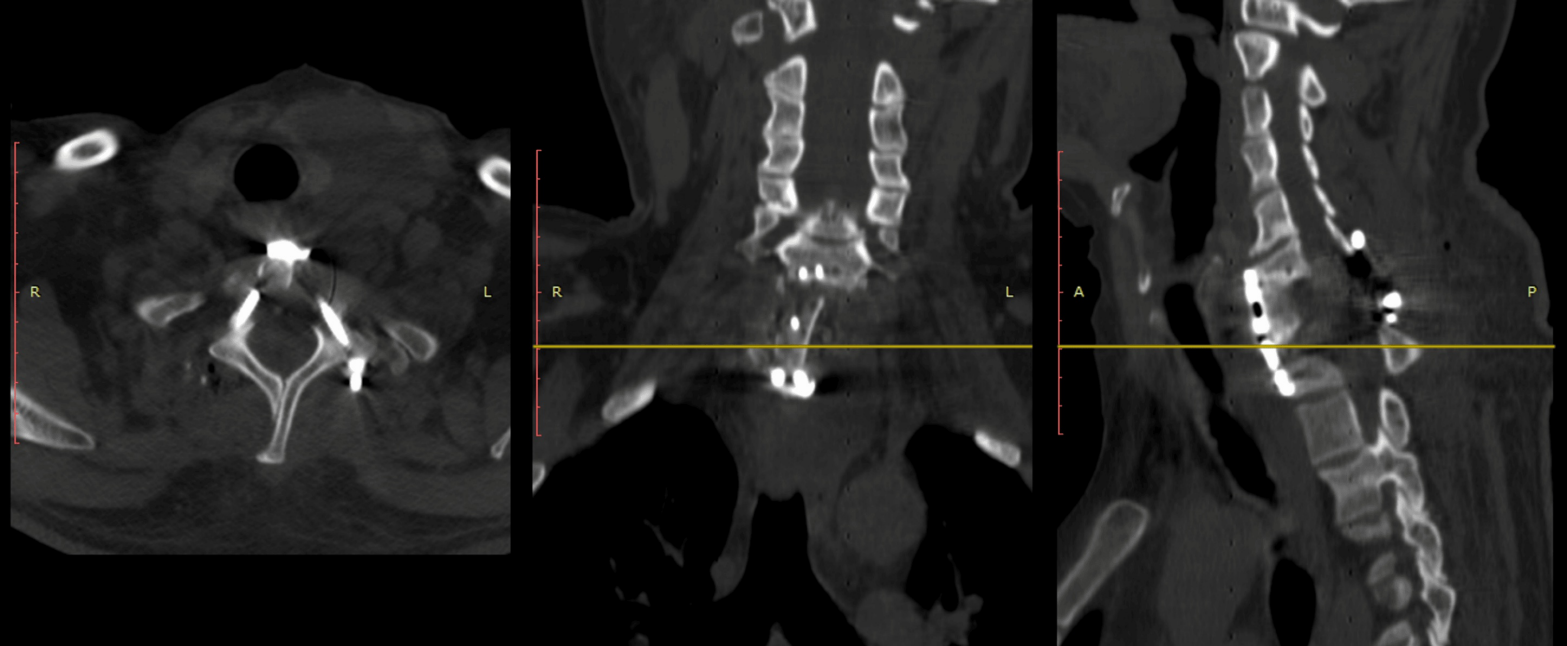
Case 4 non-contrast CT scan of the cervical spine with axial, coronal, and sagittal views of the cervical instrumentation was performed.

The patient underwent initial surgical management in the first procedure at three weeks after injury, consisting of C7 and T1 corpectomy, anterior reduction, and C6-C7-T1-T2 spondylodesis with iliac crest bone graft harvesting and application, and anterior cervical plate placement from C6 to T2. Two weeks postoperatively, transfacetary fixation of C7 and transpedicular fixation of T2 were performed, along with C7-T1 laminectomy and posterolateral arthrodesis (Figures 10, 11).

The patient developed a cerebrospinal fluid fistula through the anterior neck wound, requiring anterior revision surgery. A dural sac injury was identified and repaired. Subsequently, the patient progressed satisfactorily and was discharged from the unit seven weeks after admission. The humeral fracture was managed conservatively by the orthopedics service. 

Case 5

A 46-year-old female patient with a history of visual impairment and no chronic degenerative diseases, who suffered a fall from approximately 4 meters in height, sustaining blunt trauma to the head, thorax, and abdomen. She was initially treated at a general hospital, where she remained for seven days before being transferred to our care center.

Upon arrival, the patient was conscious and oriented, with bilateral periorbital ecchymosis present. The neck was rigid without structural or functional abnormalities. Pain was noted in the dorsolumbar region, with bilateral limitation in chest expansion and palpation. The abdomen showed no pathological signs. A Foley catheter was in place for drainage. Upper extremities were intact, and lower extremities were anatomically normal.

Neurological examination revealed intact myotomes up to T1 at 5/5 on the MRC scale. Myotomes were intact up to T11. Upper and middle abdominocutaneous reflexes were present, while the lower reflexes were abolished. Absence of the clitoridoanal reflex and anal tone was noted. Anesthesia was present from T12 distally. Lower extremities showed no motor response, and osteotendinous reflexes at L4 and S1 were absent.

**Figure 12:**
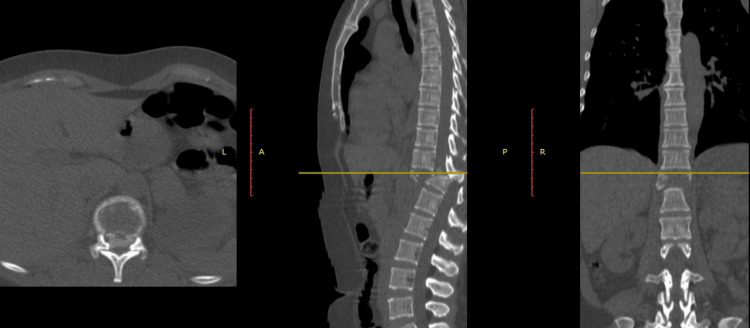
Case 5: initial CT scan with axial, coronal, and sagittal views of the injury, showing anterior displacement of the T11 vertebral body over T12.

Imaging studies upon arrival revealed a traumatic spondyloptosis of T11 over T12 with an ASIA A spinal cord injury, in addition to a clotted bilateral hemothorax (Figure 12).

**Figure 13:**
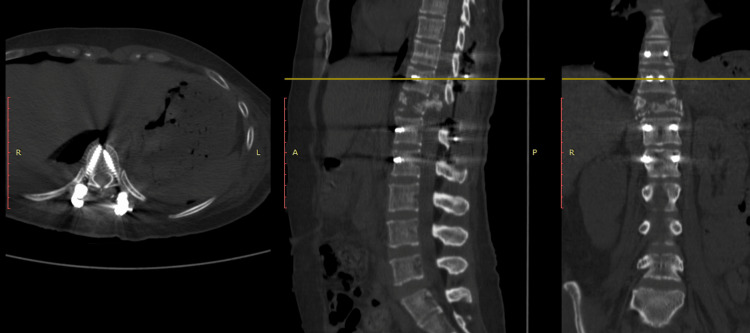
Case 5 postoperative control CT scan with axial, coronal, and sagittal views showing the transpedicular instrumentation from T10-T11 to L1-L2.

A pleurostomy tube was placed for hemothorax drainage, which was non-functional. The patient underwent posterior transpedicular instrumentation from T10-T11 to L1-L2 with posterolateral arthrodesis. During the same surgical procedure, thoracoscopy was performed for evacuation of the bilateral hemothorax (Figure 13).

The patient was discharged to the general surgery service, where she remained and was ultimately discharged 15 weeks after admission.

Results

**Figure 14:**
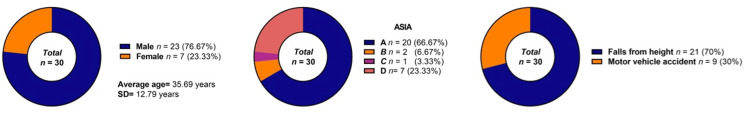
Distribution by sex, age, mechanism of injury and ASIA classification.

Thirty cases of traumatic spondyloptosis were reported. Of these, 23 were male (76.67%), and 7 were female (23.33%). The minimum age was 18 years and the maximum was 67 years, with a mean age of 35 years. Regarding the mechanism of injury, a fall from height accounted for 21 cases (70%), and a motor vehicle accident accounted for 9 cases (30%) (Figure 14).

**Figure 15:**
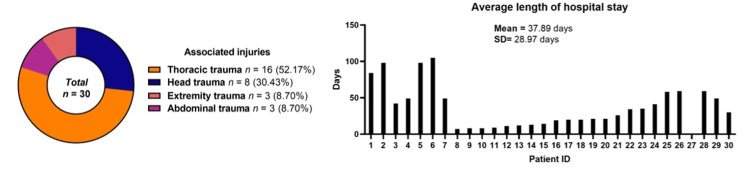
Associated injuries and average length of hospital stay.

**Figure 16:**
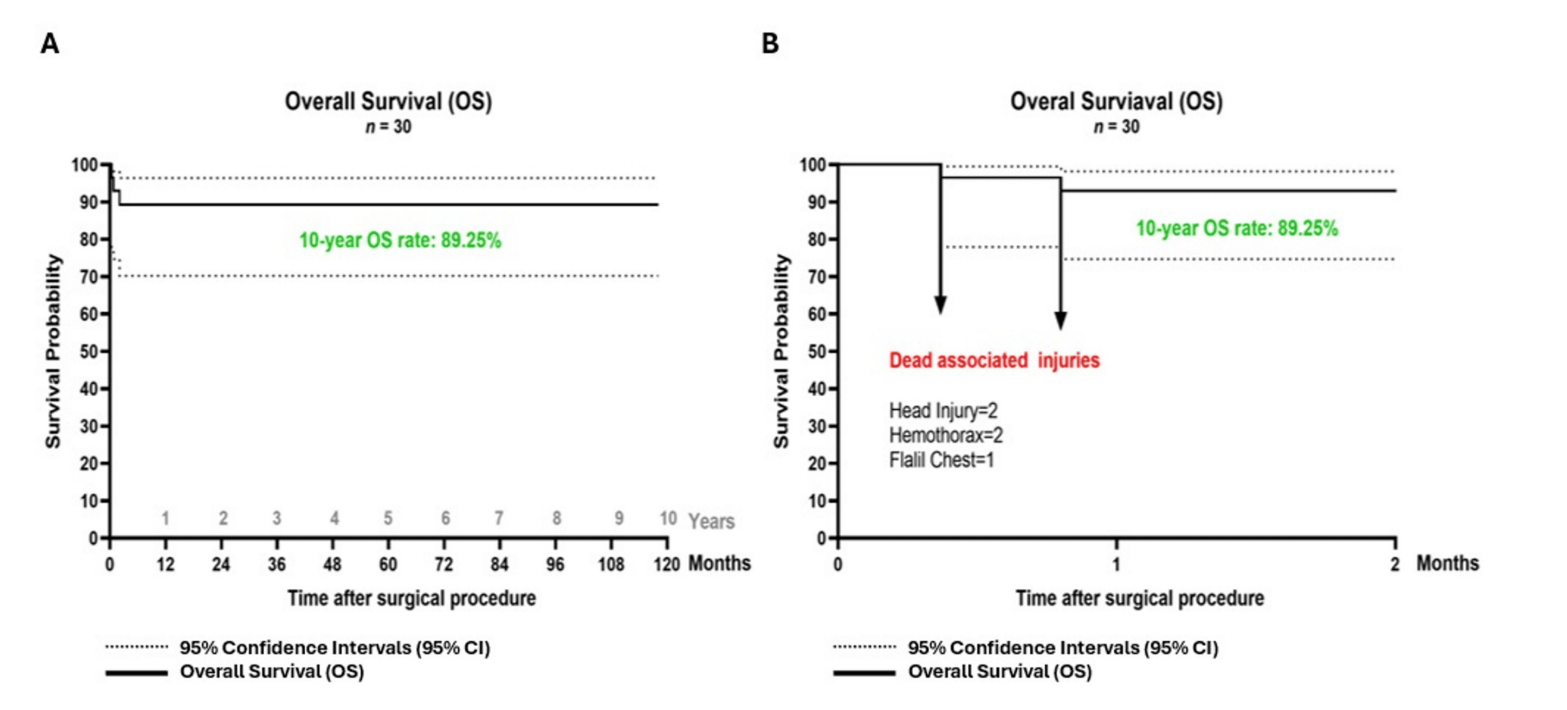
Overall survival curve. A. The plot represents the 10-year overall survival (OS) curve, where the solid line represents the overall survival probability, with the X-axis indicating post-surgery time. Dotted lines depict the 95% confidence intervals (CI), delineating the upper and lower survival limits. B. The plot represents the detailed 10-year overall survival during the first two months. Magnified view of the initial 60-day postoperative period highlighting mortality events and their associated injuries (arrows). The number of associated injuries exceeds the total number of mortality events, as multiple injuries were identified per case. The solid line represents the survival probability, while dotted lines indicate the 95% CI (upper and lower limits).

**Figure 17:**
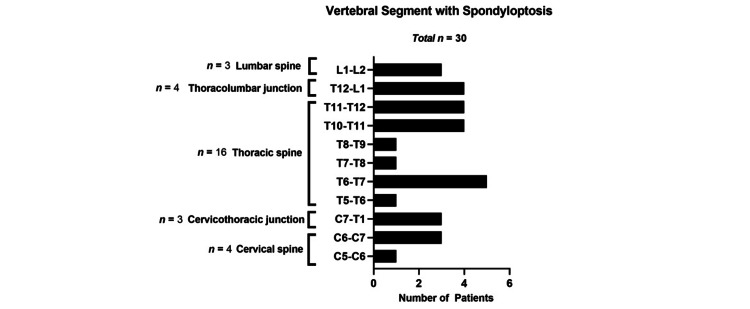
Vertebral segment with spondyloptosis.

According to the ASIA impairment scale, 20 cases (66.67%) were classified as ASIA A, 2 cases (6.67%) as ASIA B, 1 case (3.33%) as ASIA C, and 7 cases (23.33%) as ASIA D (Figure 14). Regarding associated injuries, 52.17% sustained thoracic trauma, 30.43% head trauma, 8.70% extremity trauma, and 8.70% abdominal trauma (Figure 15). The minimum hospital stay was 7 days, and the maximum was 105 days, with a mean stay of 37.89 days. (Figure 15). In our cases, 26 cases (89.25%) survived, and 4 cases (10.75%) died due to in-hospital complications. (Figure 16). The most commonly injured segments were T11-T12 and T6-T7 (Figure 17). 

## Discussion

Traumatic spondyloptosis is initially a devastating injury for both life and function due to the nature of the spinal lesion itself, associated trauma to other organs, and long-term complications. It is crucial to promptly detect associated injuries and provide timely management to enable appropriate treatment.

In our case series, we found that males were the most affected gender, comprising 76.67% of the injuries, while females accounted for 23.33%. Comparing this with a 2015 study by Akash Mishra et al., which reported 20 cases of spondyloptosis, they found that 85% of their cases were male and 15% were female [2]. The mean age in their report was 27 years, compared to 35 years in our study. Both studies agree that this type of injury predominantly affects males of working age.

In another study conducted by Chandrashekhara et al. in 2010, they reported four cases of unusual lumbar spondyloptosis. Of these four cases, one (25%) was caused by a motor vehicle accident, one (25%) by a fall from a moving vehicle, and two (50%) by a fall from a height [11]. They did not report the presence of associated injuries, in contrast to our study, nor did they report the length of hospital stay. Among the similarities found was that the most affected gender was male. A notable difference is that their cases were reported in patients in their second decade of life, unlike the mean age of 45 years reported in our study. Chandrashekhara et al. did not report any fatalities in their cases [12].

Regarding the mechanism of injury, our report identified two main causes: falls from height (70.97%) and motor vehicle accidents (29.03%), both associated with high-energy trauma. Mishra and colleagues found the same two mechanisms. The associated injuries they reported were 50% polytrauma, 20% hemothorax, pneumothorax, or both, 10% traumatic brain injury, and 10% associated blunt abdominal trauma with organ injury [2]. In our report, the most common associated injury was thoracic trauma (52.17%), followed by head trauma (30.43%), extremity trauma (8.70%), and abdominal trauma (8.70%).

The most commonly injured spinal segments were in the thoracic spine, specifically T11-T12 and T6-T7, with five cases each.

Mishra et al. found a higher incidence of injury at the thoracolumbar junction (55%), followed by the dorsal spine (35%), and 5% at the lumbosacral junction [2]. A difference in this variable is the presence in our study of four cases of cervical spondyloptosis and three cases at the cervicothoracic junction [13], whereas Mishra et al. did not report cervical injuries but did report lumbosacral injuries.

Regarding neurological injury, Mishra et al. reported that 100% of their cases had an ASIA A neurological injury [2]. In contrast, our report found 66.67% of cases were ASIA A, 6.67% ASIA B, 3.33% ASIA C, and 23.33% ASIA D. In the ASIA A cases, both in our report and the comparative study, there was no clinical improvement at follow-up.

In terms of mortality, four patients in our series died during hospitalization, representing approximately 13% of the cohort. Mishra et al. reported a higher mortality rate, with four deaths among 20 patients (25%) [2]. In both studies, mortality was primarily related to complications associated with the initial high-energy trauma and concomitant systemic injuries. Finally, comprehensive multidisciplinary management following established polytrauma treatment protocols is essential for patients with traumatic spondyloptosis. In our study, the length of hospital stay was largely influenced by the severity of associated injuries and the high-energy mechanism of trauma, which contributed significantly to morbidity and overall patient recovery. Overall, the findings of our study are largely consistent with previously published case reports, although differences in patient age, injury distribution, and associated injuries may reflect variations in trauma mechanisms and population characteristics [14-17] (Table 1).

**Table 1:** Comparative table of the findings of this report in comparison to the mentioned reports. TBI: Traumatic Brain Injury, LOS: Length of Hospital Stay, N/R: Not Reported

	No. Cases reported	Most Affected Sex	Age (median)	Most Common Injury Mechanism	Most Common Associated Injury	Most Affected Anatomical Region	LOS (median)
Present study.	30	Male	35 years	Fall from height	TBI, Hemothorax	Thoracic spine	37.89 Days
Mishra and collaborators [2].	20	Male	27 years	Fall from height	TBI, Hemothorax	Lumbar region	N/R
Chandrashekhara and collaborators [12].	4	Male	18 years	Fall from height	N/R	Lumbar region	N/R

Some limitations of this study should be acknowledged. First, the sample size was relatively small, which may limit the generalizability of the findings. Second, the study design corresponds to an observational case series, which inherently lacks a control group for comparison. Finally, the data were collected retrospectively, which may introduce potential information bias and limit the availability of certain clinical variables.

## Conclusions

Traumatic spondyloptosis is a severe spinal injury associated with significant morbidity and mortality due to the high-energy mechanisms involved and the frequent presence of associated injuries. A thorough clinical evaluation, including detailed history taking, comprehensive physical examination, and appropriate imaging studies, is essential for accurate diagnosis and management. Early multidisciplinary management aimed at addressing both the spinal injury and concomitant traumatic lesions is crucial to reduce complications, decrease mortality, and optimize patient outcomes. Prompt treatment may also contribute to shorter hospital stays and improve the likelihood of functional recovery and social reintegration.
